# Clinical profile of autoimmune nodopathy with anti‐neurofascin 186 antibody

**DOI:** 10.1002/acn3.51775

**Published:** 2023-04-14

**Authors:** Bingyou Liu, Lei Zhou, Chong Sun, Longjie Wang, Yongsheng Zheng, Bin Hu, Kai Qiao, Chongbo Zhao, Jiahong Lu, Jie Lin

**Affiliations:** ^1^ Department of Neurology, Huashan Hospital Fudan University Shanghai China; ^2^ Electron Microscopy Center, Department of Nephrology, Huashan Hospital North Branch Fudan University Shanghai China; ^3^ Department of Radiology, Huashan Hospital Fudan University Shanghai China

## Abstract

**Objective:**

Nodal/paranodal autoantibodies identified a group of peripheral neuropathies independent from chronic inflammatory demyelinating polyneuropathy (CIDP). However, nodopathy with antibody against neurofascin 186 (NF186) was rarely reported. We presented a cohort of patients with anti‐NF186 antibody and described the clinical profile of them.

**Methods:**

In this retrospective study, 195 patients diagnosed with CIDP and immune mediated idiopathic neuropathies were enrolled. Cell‐based assay was used to screen anti‐NF186 and anti‐NF155 antibodies in serum samples. Teased‐fiber immunofluorescence were used as a confirmatory assay. Clinical data of seropositive patients were collected and analyzed.

**Results:**

Among the patients with anti‐NF186 antibody, seven patients (58.3%) presented acute or subacute disorder onset. Four patients (33.3%) were found to have asymmetric weakness or numbness. Distal weakness and/or numbness was the core feature. Sensory ataxia, tremor and central nervous system demyelination were rarely observed. Nerve conduction studies revealed predominant demyelinating with/without axonal loss. Brachial plexus MRI was normal in the majority of patients (6/7, 85.7%). Five patients (5/9, 55.6%) showed response to intravenous immunoglobulin. Eight patients (8/10, 80.0%) improved after corticosteroids. All patients (3/3,100%) responded to rituximab.

**Interpretation:**

In the study, we depicted the clinical profile of nodopathy with anti‐NF186 antibody. The diversity of clinical features, electrophysiology results and pathological findings was specific in nodopathy with anti‐NF186 antibody. Screening of autoantibody against NF186 in acute‐onset neuropathy is recommended.

## Introduction

Autoantibodies against proteins in the nodal and paranodal regions were found in a subset of patients with peripheral neuropathy, including anti‐contactin 1 (CNTN1), anti‐neurofascin 155 (NF155), anti‐contactin‐associated protein 1 (Caspr1) and anti‐neurofascin 186 (NF186).^1^, ^2^, ^3^, ^4^, ^5^ These autoantibodies distinguished neuropathies with special clinical features, which were further defined as autoimmune nodopathies independent from chronic inflammatory demyelinating polyneuropathy (CIDP).^6^

Neurofascin 186 is a cell adhesion molecule containing six immunoglobulin‐like domains, four fibronectin type III domains and one mucin domain. Located on axons, it interacts with neuronal cell adhesion molecule (NrCAM) and gliomedin, then, is further linked to cytoskeleton by ankyrin‐G. It is essential for the clustering of voltage‐gated sodium channels at nodes of Ranvier.^7^, ^8^, ^9^

Nodopathy with anti‐NF186 antibody was rarely reported. Several cases presented it as a subacute onset disorder with distal acquired demyelinating syndrome, sensory ataxia, cranial nerve involvement, a good response to steroids and intravenous immunoglobulin (IVIG).^4^ However, the clinical features of nodopathy with anti‐NF186 antibody are still not well elaborated. It is difficult to distinguish nodopathy with anti‐NF186 antibody from those with anti‐NF155 antibody. Herein, we reported a cohort of patients with anti‐NF186 antibody (anti‐NF186+) and compared their clinical features with patients with anti‐NF155 antibody (anti‐NF155+).

## Methods

### Patients and sera

Sera samples from 195 patients diagnosed with definite and probable CIDP by EFNS/PNS 2010 criteria^10^ and immune‐mediated idiopathic neuropathies were obtained and stored at −80°C. They were accumulated between 2018 and 2021 in Huashan Hospital, Fudan University. Sera samples from 13 patients with Charcot–Marie–Tooth disease and two healthy adults were utilized as controls. Cell‐based assay of anti‐NF155 and anti‐NF186 antibodies were performed in 195 samples. Clinical data were retrospectively collected from hospital database and follow‐up records. Treatment outcome was defined as favorable in patients whose Modified Rankin scale (mRS) scores were decreased by one or more grades after treatment.

Written informed consent was obtained from each participant. The study protocol was approved by the ethics committee of Huashan Hospital, Fudan University.

### Cell‐based assay

Human NF186 (NM_001005388.3) and NF155 (NM_001160331.2) cDNA was subcloned into peGFP‐N1 (Umibio biotec, Shanghai China) using a site‐directed mutagenesis kit. HEK293 cells (Umibio Biotec, Shanghai, China) were plated onto poly‐L‐lysine coated glass coverslips in fetal bovine serum‐supplemented Dulbecco modified Eagle culture media (DMEM). The peGFP‐N1 vector containing cDNA encoding human NF186 and NF155 was diluted in Opti‐MEM I Reduced Serum Medium (Thermo Fisher) and transfected with Lipofectamine 2000 (Thermo Fisher) at 60 to 70% cell confluence. The day after transfection, serum samples were diluted with DMEM (1:10 ~ 1:1000) and incubated in the wells. Then, the cells were fixed with 4% paraformaldehyde and blocked by 3% BSA. For sample screening, Alexa 568‐conjugated goat anti‐human IgG (H + L, 1:1000, Invitrogen) and Alexa 594‐conjugated IgM (μ, 1:2000, Invitrogen) were diluted with 1%BSA and incubated overnight. For isotype identification, mouse anti‐human IgG1 to 3 (1:500, Sigma), and IgG4 (1:100, Sigma) were diluted with 1% BSA and incubated overnight and then Alexa Fluor 555‐conjugated goat anti‐mouse IgG (H + L, 1:1000, Invitrogen) were diluted with 1% BSA and incubated for 1 h. After several washes with PBS, the coverslips were fetched and mounted onto slides with DAPI Fluoromount‐G (Southern Biotech) for examination under the fluorescence microscope (Olympus BX60). Colocalization of NF186 (green fluorescence) and anti‐NF186 antibody (red fluorescence) were defined as positive. Cell‐based assays were repeated at a different time point for each sample, results of which were read out by identical physician. Final decisions were made based on coincident results.

### Immunofluorescence assay on teased fibers

The sciatic nerve from adult Lewis rat was dissected and fixed in 4% paraformaldehyde at 4°C overnight. Teased fibers were prepared by gently teasing the nerve fibers with two fine forceps on a glass slide immersed in PBS. The fibers were air‐dried and permeabilized with 0.3% TritonX‐100 in PBS for 30 min, then blocked with PBS containing 10% normal goat serum and 0.1% TritonX‐100 for 1 h at room temperature. The fibers were then incubated with rabbit anti‐Caspr1 (1:500, Invitrogen) and patients' sera (1:40) for 1 h at room temperature. After three washes with PBS, the fibers were incubated with Alexa Fluor 488‐conjugated goat anti‐rabbit IgG (H + L, 1:1000, Invitrogen) and Alexa 568‐conjugated goat anti‐human IgG (H + L, 1:1000, Invitrogen). After three washes with PBST (Tween 20, 0.001%), the slides were mounted with mounting medium (Beyotime, Shanghai, China) for examination under the fluorescence microscope (Olympus BX60).

### Laboratory examinations

Laboratory tests including routine blood, biochemical test, blood glucose, monoclonal immunoglobulin analysis by electrophoresis, combined blood and urine analysis for immunoglobulin free light chain, folic acid and vitamin B12 level, cytology and biochemical tests of cerebrospinal fluid were examined. Additional serum autoimmune biomarker tests including antinuclear antibodies, anti‐dsDNA antibodies, anti‐extractable nuclear antigens spectrum antibodies, anti‐neutrophil cytoplasmic antibodies, anti‐mitochondrial antibodies, anti‐thyroperoxidase antibodies, anti‐thyroglobulin antibodies, and anti‐ganglioside antibodies were also performed.

Sural nerve biopsy was performed in one patient (P12). The semi‐thin sections were stained with toluidine blue. Hematoxylin and eosin (H&E), modified Gomori trichrome, and immunohistochemical staining of CD3, CD4, CD20, and CD68 were routinely stained. Longitudinal semi‐thin sections were observed under electron microscopy to study the morphological changes of node of Ranvier.

### Electrophysiology

Nerve conduction velocity (NCV) and needle electromyography (EMG) studies were completed at room temperature. Electrophysiological diagnosis of CIDP were made based on EFNS/PNS 2010 criteria.

### Brachial plexus MRI

Brachial plexus MRI were performed on a 3.0 T Siemens Magnetom Verio MR scanner. The parameters were: repetition time at 2880 ms, echo time at 80 ms, field of view at 220 × 220 mm, matrix at 320 × 240. T2 short‐tau inversion recovery sequence (STIR) or CUBE STIR images were acquired on the coronal plane, in which diameters of nerve roots were measured. Radiological diagnosis was performed by one neuroradiologist who was blinded to the diagnosis.

### Statistical analysis

Statistical analyses were conducted with SPSS version 21.0 software (SPSS Inc., Chicago IL, USA). Data were presented as medians with ranges or means ±1 standard deviation (SD) according to their distribution. Fisher's exact‐probability test, Chi‐square test, Student's *t* test or Mann–Whitney U test were used. Statistical significance was set at *p* < 0.05.

## Results

### Antibody identification

**Figure 1:**
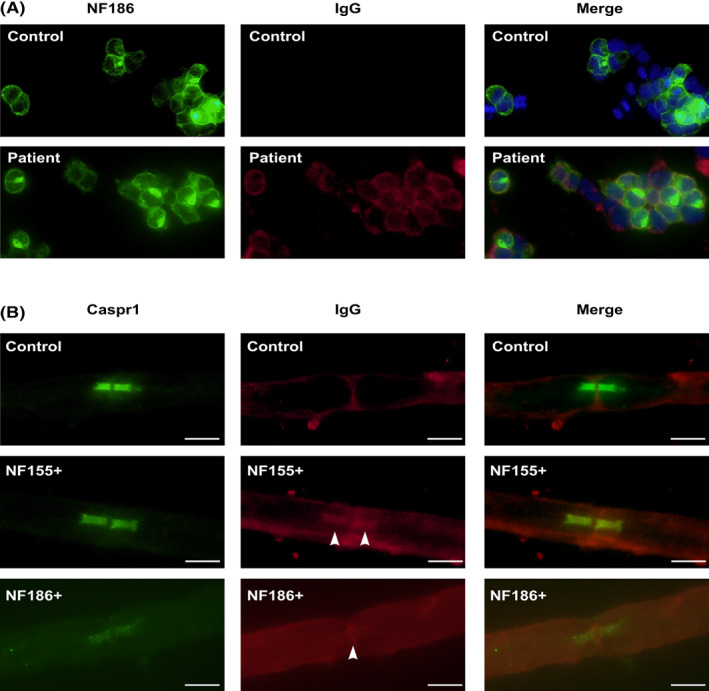
Example of cell‐based assay and teased‐fiber assay of anti‐neurofascin antibody. (A) Cell‐based assay of negative control and anti‐NF186+ patient. NF186 (green fluorescence) colocalized with anti‐NF186 antibody (red fluorescence) in P8, which indicated the presence of anti‐NF186 IgG. Nuclei of HEK cells were shown in blue fluorescence with DAPI. (B) Teased fiber assay of negative control, anti‐NF155+ and anti‐NF186+ patient. Paranodal regions were shown by Caspr1 (green fluorescence), serum from anti‐NF155+ or anti‐NF186+ patients showed paranodal or nodal binding, respectively (red fluorescence, indicated with arrow head).

Among 195 patients, there were 13 patients with anti‐NF186 antibody (anti‐NF186+) and 20 patients with anti‐NF155 antibody (anti‐NF155+). All 13 patients with anti‐NF186 antibody had anti‐NF186 IgG (Fig. 1A), no sample was found with co‐occurrence of anti‐NF186 and anti‐NF155 antibodies. P2 was found with both IgG and IgM binding. There were four samples (from P1, P3, P8, and P12) had detectable IgG isotypes, while isotypes were undetectable in remaining samples. Sera from all seropositive patients showed reactivity in either nodal or paranodal regions of the teased fibers (Fig. 1B).

### Clinical characteristics of patients with anti‐NF186 antibody

In 13 patients with anti‐NF186 antibody, 12 patients presented with a clinical phenotype of polyneuropathy. The other patient (P13) was only found with cranial nerve involvement and diagnosed with immune mediate idiopathic neuropathy. All patients presented either chronic (7/13, 53.8%) or relapsing (6/13, 46.2%) course. There was no patient in our study showed disautonomia or nephrotic syndrome.

In the polyneuropathy cohort, nine patients (9/12, 75.0%) with anti‐NF186 antibody were male. The mean onset age was 43.4 ± 20.7 yo. An acute or subacute onset pattern was observed in seven patients (7/12, 58.3%). Eight patients developed symmetric weakness. The asymmetric weakness of the other four patients was mainly observed at disease onset. Weakness was identified in both proximal and distal extremities. One patient had cranial nerve involvement. Detailed data were shown in Table S1.

Clinal features of anti‐NF186+ nodopathy and anti‐NF155+ nodopathy were shown in Table 1. Compared to the anti‐NF155+ group, anti‐NF186+ patients had an older onset age. More anti‐NF186+ patients suffered an acute/subacute disease onset. Weakness in both proximal and distal limbs were more prominent in anti‐NF186+ patients. Anti‐NF186+ patients had fewer vibration sensation disturbances. Sensory ataxia and tremor of extremities was rarely observed in anti‐NF186+ patients. In contrast, this is the significant feature in anti‐NF155+ patients. Auxiliary examination results and treatment responses were shown in Table 2. CSF protein level was lower in anti‐NF186+ patients. Steroids treatment showed better response in anti‐NF186+ patients. Rituximab was effective in patients with either anti‐NF155 or anti‐NF186 antibody.

**Table 1:** Clinical feature comparison between anti‐NF186+ and anti‐NF155+ polyneuropathy.

	Anti‐NF186 (*N* = 12^1^)	Anti‐NF155 (*N* = 20)	*p* value
Detection rate	12/195, 6.2%	20/195,10.3%	‐
Age at onset (year)	43.4 ± 20.7	26.0 ± 14.7	0.010
Disease course (month), median (range)	5.5 (0.5–72)	9.5 (2–86)	>0.05
Sex, male, *n* (%)	9 (75.0)	18 (90.0)	>0.05
Prodrome, *n* (%)	3 (25.0)	4 (20.0)	>0.05
Acute/Subacute onset, *n* (%)	7 (58.3)	4 (20.0)	0.053
Weakness, *n* (%)			
UL proximal	7 (58.3)	3 (15.0)	0.018
UL distal	11 (91.7)	11 (55.0)	0.050
LL proximal	10 (83.3)	8 (40.0)	0.028
LL distal	10 (83.3)	18 (90.0)	>0.05
Symmetric	8 (66.7)	19 (95.0)	0.053
MRC muscle strength score of proximal limb <4	5 (41.7)	0 (0)	0.004
Sensory disturbance, *n* (%)			
Superficial sensation	9 (75.0)	15 (75.0)	>0.05
Vibration sensation	6 (50.0)	20 (100)	0.001
Symmetric	9 (75.0)	20 (100)	0.044
Sensory ataxia	4 (33.3)	19 (95.0)	0.0006
Tremor, *n* (%)	1 (8.3)	15 (75.0)	0.0002
Cranial nerve involvement, *n* (%)	1 (8.3)	3 (15.0)	>0.05
CNS demyelination, *n* (%)	1 (8.3)	1 (5.0)	>0.05
Intensive care unit, *n* (%)	1 (8.3)	0 (0)	>0.05
Modified Rankin Scale, median (range)	3 (1–5)	3 (1–4)	>0.05

UL: upper limb; LL: lower limb.

^1^ P13 was excluded from the comparison.

**Table 2:** Auxiliary examination results and treatment responses of anti‐NF186+ and anti‐NF155+ polyneuropathy.

	Anti‐NF186 (*N* = 12^1^)	Anti‐NF155 (*N* = 20)	*p* value
CSF protein, median (range)^1^	773 (270–7102)	3144 (855–15000)	0.026
Nerve conduction study, *n* (%)^2^			
Demyelinating predominant	8 (66.7)	18 (100)	0.018
Accompanied axon loss	6 (50.0)	18 (100)	0.002
Conduction blocks	2 (16.7)	5 (27.8)	>0.05
Temporal dispersion	2 (16.7)	2 (11.1)	>0.05
Axonal loss predominant	3 (25.0)	0 (0)	0.054
Brachial plexus abnormal, *n* (%)	1/7 (14.3)	15/16 (93.8)	0.0005
Good response, *n* (%)			
IVIG	5/9 (55.6)	1/9 (11.1)	>0.05
Steroids	8/10 (80.0)	4/19 (21.1)	0.005
PE/DFPP	3/4 (75.0)	5/8 (62.5)	–
Rituximab	3/3 (100)	12/12 (100)	–
Other immune suppressors	0/1 (0)	1/5 (20.0)^3^	–

IVIG: intravenous immunoglobulin; PE: plasma exchange; DFPP: double‐filtration plasmapheresis.

^1^ Four NF155+ patients not done.

^2^ One NF186+ patient presented normal nerve conduction study, 2 NF155+ patients not done.

^3^ One patient achieved remission with oral azathioprine.

### Electrophysiology

**Figure 2:**
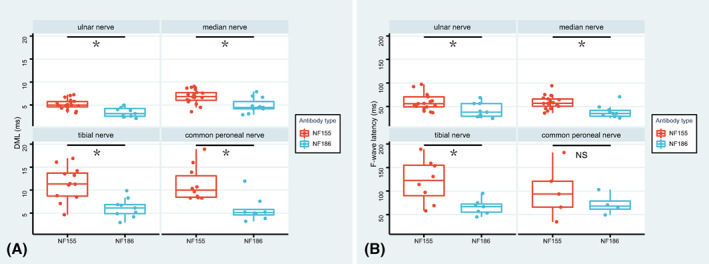
Distal motor latency and F‐wave latency of anti‐NF186+ and anti‐NF155+ patients. (A). Comparation of distal motor latency of ulnar nerve, median nerve, tibial nerve and common peroneal nerve between anti‐NF186+ and anti‐NF155+ patients; (B) Comparation of F‐wave latency of ulnar nerve, median nerve, tibial nerve and common peroneal nerve between anti‐NF186+ and anti‐NF155+ patients. (Patient 13 was excluded for solely cranial nerve involvement; *p < 0.05; NS = no statistical significance.)

Nerve conduction studies revealed demyelinating and/or axonal loss features in anti‐NF186+ patients. All anti‐NF155+ patients were found with demyelinating and accompanied axonal loss. However, it was only found in 50% patients with anti‐NF186 antibody. Three anti‐NF186+ patients were found with axonal loss only. Distal motor latency and F‐wave latency of anti‐NF186+ patients were shorter than anti‐NF155+ patients in most tested nerves (Fig. 2).

### Nerve biopsy

**Figure 3:**
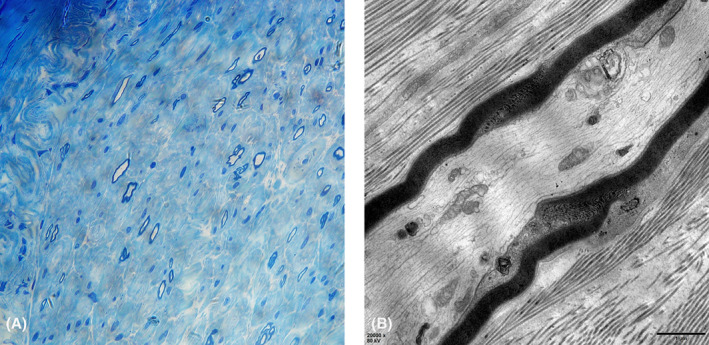
Sural nerve biopsy of P12. (A) Transverse sections stained with toluidine blue. Decrease of the number of myelinated fibers and regeneration were observed without cellular infiltration or onion‐bulb formation; (B) Longitudinal section under electron microscopy. Paranode loops fused and occupied the node and paranode region. Scale bar = 1 μm.

Sural nerve biopsy was performed in P12. In semi‐thin sections, the density of large and small myelinated fibers severely reduced (Fig. 3A). CD3, CD4, CD20 and CD68 staining were negative. In electron microscopy of longitudinal sections, it was observed that the flanking paranodes extended to the node. The terminal loops once separated by node fused to form a “myelin sheath sleeve”, approximately 3 μm in length, and occupied the node and paranode region (Fig. 3B).

### Brachial plexus MRI findings

**Figure 4:**
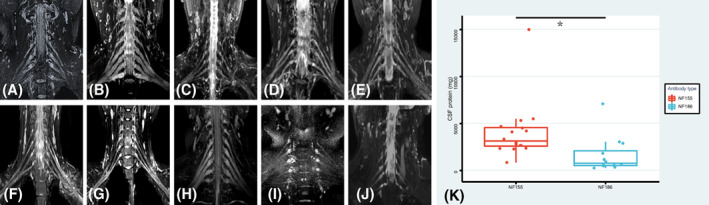
Brachial plexus MRI of anti‐NF186+ patients and CSF protein level of anti‐NF186+ and anti‐NF155+ patients. (A) Brachial plexus MRI of healthy control; (B) Typical brachial plexus MRI of anti‐NF155+ neuropathy; (C–J) Brachial plexus MRI of P1, P2, P3, P4, P7, P8, P12 and P13 with anti‐NF186 antibody. (K) Comparation of CSF protein level between anti‐NF186+ and anti‐NF155+ patients (*p < 0.05).

Brachial plexus MRI was performed in seven anti‐NF186+ patients. Only P8 showed symmetric and diffuse nerve root enlargement. Nerve root enlargement was rare in anti‐NF186+ patients, but prominent in anti‐NF155+ patients (Fig. 4). A total of 15 patients (93.8%, 15/16) with anti‐NF155 antibody in our cohort showed diffusing nerve root enlargement.

## Discussion

Neuropathies with nodal/paranodal antibodies were originally classified as CIDP according to EFNS/PNS 2010 criteria. However, the pathogenic mechanism and clinical characteristics of these patients were different from CIDP as revealed in further studies. Hence, neuropathies with nodal/paranodal antibodies were defined as autoimmune nodopathies independent from CIDP in 2021 EAN/PNS criteria.

In this study, the detection rate of anti‐NF186 antibody was relatively higher. Firstly, this may be the result of sample collection. The collected samples were mainly from our inpatients, most of which had prominent clinical manifestations or resembled the phenotype of GBS or CIDP. Thus, there was the possibility that some patients with milder involvement and those showed predominately axon damage were left out. Then, the racial factor should be taken into consideration. Patients with anti‐nodal/paranodal antibodies from different population showed some phenotype discrepancy and the detection rate was also different. For instance, according to previous reports from European institutes, anti‐NF155+ patients could manifest with prominent motor involvement, even severe quadriplegia, but this was rarely reported in Asian patients from China, Japan or Malaysia, where the patients showed severer sensory involvement.

Several notable clinical features of anti‐NF186+ nodopathy were observed. First, most patients experienced acute/subacute onset disease courses, either a “GBS‐like” acute onset or acute attacks in the chronic course. In those patients with acute onset, the early motor and sensory involvements were easily misdiagnosed as GBS and consequently administrated IVIG treatment. However, the response was poor. Hence, we recommend anti‐NF186 antibody screening in patients with the suspicion of acquired acute‐onset neuropathy. Second, the weakness was not only in the distal extremities but also in the proximal limbs in anti‐NF186+ patients. Moreover, the weakness in the proximal limbs was more prominent in anti‐NF186+ patients which was similar to classic CIDP. But distal weakness was found spontaneously when proximal weakness involved or rapidly progressed. In addition, asymmetric involvement was common among patients with anti‐NF186 antibody. It should be distinguished from focal CIDP or Lewis‐Sumner syndrome. Third, several patients showed disturbance of vibration sensation and sensory ataxia. However, it was less observed in anti‐NF186+ patients. These phenotype differences may be associated with IgG subtype distribution of the antibody. IgG subtypes were all screened in our patients. IgG3 and/or IgG4 subtypes were reported in previous studies.^4^, ^5^, ^11^ But in our study, only one patient showed IgG4 predominance. Nine patients were unable to further identify IgG subtypes by CBA (See in Table S1). The diversity of the distribution of Ig isotypes and IgG subtypes in nodo/paranodal neuropathy may relate to the characteristics of antibodies, the stage of the disease and treatments.^5^ The mechanism was unclarified.

CNS demyelination was rarely seen in our study. P2 was the only patient with CNS demyelinating clues revealed by MRI and had concomitant lung cancer along with serum CV2 antibody. Coexistence of dysimmune diseases in anti‐NF186+ patients was reported. Screening of anti‐NF155 and anti‐NF186 antibodies was recommended for patients with the suspicion of combined central and peripheral demyelination. It also deserves attention that patients with anti‐NF186 autoantibody can manifest with solely cranial nerve involvement, which was rarely reported in previous studies of nodal/paranodal neuropathies. However, cranial nerve involvement showed no difference between anti‐NF186+ and anti‐NF 155+ groups.

Nerve conduction studies of anti‐NF186+ patients were heterogeneous and scattered in a spectrum from demyelinating to axonal loss. Comparing with anti‐NF155+ patients, anti‐NF186+ patients had shorter distal motor latency and F‐wave latency, which may indicate a milder disruption of nerve conduction in distal and proximal segments. Most anti‐NF186+ patients showed demyelinating with or without axonal loss. As an axonal adhesion molecule, NF186 functions in the clustering of sodium channels. The disruption of NF186 could influence the stability and function of nodal sodium channels. It rendered saltatory conduction failure, presenting as “demyelinating features” including prolonged distal potential latencies, conduction blocks, and temporal dispersion in nerve conduction study. On the other hand, NF186 was also associated with axonal cytoskeleton proteins and the severe disruption may cause axonal loss directly.^8^

Interestingly, in this study, we observed the pathological feature of anti‐NF186+ nodopathy. NF186 was an adhesion molecule essential for node assembly. The dysfunction of NF186 can render malformation of node. In the NF186 knock‐out animal model, it was found that ablation of NF186 caused nodal disorganization with paranode “invasion” to the node region. The paranode loops from one side showed a tendency of climbing up to the other side to form an overlapping structure.^12^, ^13^ Similar phenomenon was also observed in one CIDP patient with anti‐NF186 IgG3.^11^ But these changes may both be the premature stages of the structural evolution. In our study, this disorganization was observed in an advanced stage. The flanking paranode loops fused and formed an overlapping special structure. It was named “myelin sheath sleeve” which obliterated the node structure and occupied the node and paranode region. We suggest that this phenomenon could be attributed to the chronic pathological processes caused by anti‐NF186 antibody. However, the exact mechanism of the formation of this structure remains to be elucidated.

Nerve root symmetric enlargement, T2 hyperintensity and abnormal enhancement in MRI was a prominent feature of anti‐NF155+ nodopathy.^14^ In our study, brachial plexus MRI abnormality was only found in one anti‐NF186+ patient. The nerve root abnormality may reflect the disruption of blood‐nerve barrier.^15^ The CSF protein level in anti‐NF186+ patients was lower than that in anti‐NF155+ group. Shorter F‐wave latencies was also found in anti‐NF186+ patients. Hence, we inferred that the disruption of blood‐nerve barrier may be milder in anti‐NF186+ patients than in anti‐NF155+ patients in the proximal nerve segment. However, the mechanism of branchial plexus MRI abnormality in anti‐NF186+ antibody patients is unclear.

Treatment with steroids favorably affected patients in our cohort. Eight of ten patients received intravenous steroids treatment showed mRS score improvement. Anti‐NF186+ patients presented rapid electrophysiology recovery as the result of an excellent treatment response. P11 mainly manifested with subacute weakness and numbness of four limbs. He achieved rapid remission with steroids and IVIG. The nerve conduction study was normal after initiation of treatment for 15 days.

IVIG was reported to be ineffective in previous clinical studies of autoimmune nodopathy, especially those with anti‐NF155 or anti‐CNTN1 antibodies. The total efficiency was reported to be less than 40%.^2^, ^16^, ^17^, ^18^ The unique features of IgG4 may render the lack of ability to activate a conventional complement‐mediated or cellular immune response and the difficulty for antibody binding by IVIG.^19^ This may account for the IVIG resistance and the theory was supported by pathologic findings.^20^, ^21^, ^22^ In our study, B cell depletion therapy, namely Rituximab, was effective in all patients. Further investigation could verify the long‐term effect of Rituximab.

Several researches indicated that pan‐neurofascin antibodies identify a severe yet treatable neuropathy.^23^, ^24^, ^25^ However, anti‐pan‐neurofascin antibody was not detected in our cohort. It may be the result of limited sample size. Patients with anti‐NF186 antibody shared some characteristics with patients with anti‐pan‐neurofascin antibody, especially acute or “GBS” like onset. Yet they suffered a milder deterioration, showed better response to initial treatment and presented either a chronic or relapsing rather than a monophasic disease course.

It is inevitable there are some limitations in this study. First, the sample size was small due to the rarity of this disease. Data from larger patient cohorts are required to confirm our findings. Second, this is a cross‐sectional study which was limited by a lack of follow‐up results.

In conclusion, our study summarized the clinical profile of patients with anti‐NF186 antibody. They presented more complicated features compared to previous studies and were distinguished with anti‐NF155+ nodopathy. Interesting pathological changes were observed in the node of Ranvier, which might shed light on the pathophysiology of the disease and help further elucidate the mechanism of disease evolution.

## Funding Information

The authors did not receive support from any organization for the submitted work.

## Conflict of Interest

The authors declare that they have no conflict of interest.

## Author contributions

Jie Lin, Chongbo Zhao and Jiahong Lu contributed to the conception and design of the study; Bingyou Liu, Lei Zhou, Chong Sun, Longjie Wang, Yongsheng Zheng, Bin Hu and Kai Qiao contributed to the acquisition and analysis of data; Bingyou Liu and Jie Lin contributed to the drafting of the manuscript and figures; all authors contributed to revising the manuscript critically for important intellectual content.

## Supporting information


Table S1.
