# Supplementary material for: Clinical profile of autoimmune nodopathy with anti‐neurofascin 186 antibody
Source: Ann Clin Transl Neurol. 2023 Apr 14;10(6):944–52. doi: 10.1002/acn3.51775 (PMC10270277; doi:10.1002/acn3.51775)
Supplement: Supplementary file 1 — Table S1. [file ACN3-10-944-s001.docx]

| **Supplementary Table 1 Detailed clinical characteristics of patients with anti-NF186 antibody** | | | | | | | | | | | | | | | | | | | | |
| --- | --- | --- | --- | --- | --- | --- | --- | --- | --- | --- | --- | --- | --- | --- | --- | --- | --- | --- | --- | --- |
| Number | Gender | Age at onset  (year) | Onset | Disease course^a^  (Month) | Prodrome | Limb weakness | Sensation disturbance | | Symmetry | Sensory ataxia | Cranial nerve involvement | Tremor | mRS | CSF protein  (mg/L) | Other immune  dysfunction clues | Electrophysiology | Brachial plexus MRI | CNS demyelination | Antibody isotype |  |
|  |  |  |  |  |  |  | Superficial | Vibration |  |  |  |  |  |  |  |  |  |  |  |  |
| P1 | M | 40 | Subacute | 24 | No | LLL distal 3+; RLL proximal 4+, distal 3 | Yes | No | No | No | No | No | 3 | 3051 | ATPO（+）,ATG（+）, Ro 52 (+) | Demyelinating, accompanied axon loss, temporal dispersion | Normal | No | IgG (IgG1) |  |
| P2 | M | 57 | Chronic | 7 | No | UL proximal 4-, distal 3; LL proximal and distal 1 | Yes | Yes | Yes | No | No | No | 5 | 928 | ANA 1:100, CV2(+) | Demyelinating, accompanied axon loss | Normal | Yes | IgM  IgG |  |
| P3 | F | 62 | Acute | 3 | No | UL proximal and distal 4; LL proximal and distal 3+ | Yes | Yes | Yes | Yes | No | No | 4 | 1831 | No | Demyelinating, accompanied axon loss | Normal | No | IgG (IgG2) |  |
| P4 | M | 20 | Chronic | 12 | No | LUL distal 4 | No | No | No | No | No | No | 1 | 543 | No | Demyelinating, accompanied axon loss, temporal dispersion | Normal | No | IgG |  |
| P5 | M | 30 | Subacute | 5 | Upper respiratory tract infection | UL proximal 4，distal 3-; LL proximal 4-，distal 3 | No | No | Yes | No | No | No | 3 | 1178 | No | Demyelinating,  conduction blocks | ND | No | IgG |  |
| P6 | M | 34 | Acute^b^ | 0.5  (limb weakness 3 months to peak) | Upper respiratory tract infection | UL proximal 4，distal 4+; LL proximal 3，distal 1 | Yes | No | Yes | No | Dysphonia，bilateral peripheral facial and lingual paralysis | No | 5 | 355 | ATPO（+）, ATG（+）, ANA 1:100,  pANCA（+） | Axonal loss | ND | No | IgG |  |
| P7 | F | 17 | Subacute | 1, (Symptom lasted for 3 months after treatment) | No | RUL distal 3 | No | No | No | No | No | No | 1 | 270 | No | Demyelinating, accompanied axon loss,  conduction blocks | Normal | No | IgG |  |
| P8 | M | 13 | Acute | 5 | Diarrhea | UL proximal 4, distal 2; LL proximal 4, distal 2 | Yes | Yes | Yes | Yes | No | No | 4 | 2898 | ATPO（+）, ATG（+）, ANA 1:100,  AMA M2（+） | Axonal loss | Abnormal | No | IgG (IgG1、IgG2、IgG3) |  |
| P9 | M | 77 | Chronic | 6 | No | UL proximal 4，distal 2; LL proximal 3，distal 1 | Yes | Yes | Yes | Yes | No | No | 4 | 7102 | No | Demyelinating, accompanied axon loss | ND | No | IgG |  |
| P10 | F | 55 | Chronic | 36 | No | UL distal 4; LL proximal and distal 5- | Yes | No | Yes | No | No | Yes | 3 | 618 | No | Demyelinating | ND | No | IgG |  |
| P11 | M | 62 | Subacute | 2 | No | LUL proximal 4，distal 4-; RUL proximal and distal 5-; LLL distal 4; RLL proximal and distal 5- | Yes | Yes | No | No | No | No | 3 | 547 | ATPO（+）, ANA 1:100 | Normal | ND | No | IgG |  |
| P12 | M | 51 | Chronic | 72 | No | UL distal 3; LL proximal 5-，distal 2 | Yes | Yes | Yes | Yes | No | No | 3 | 500 | No | Axonal loss | Normal | No | IgG (IgG3、IgG4） |  |
| P13 | M | 44 | Acute | 10 | Pharyngalgia and  hoarseness | No | No | No | No | No | Dysphagia, ipsilateral peripheral lingual paralysis and extraocular muscles paralysis | No | 2 | 207 | No | Left blink reflex impairment | Normal | No | IgG |  |
| a. Disease course was defined as the time span from the first symptom onset to the first visit to our institute.  b. Specially, patient 6 was defined as acute onset though the limb weakness progressed to 3 months for acute respiratory function impairment and ICU admission.  UL: upper limb; LL: lower limb; LUL: left upper limb; RUL: right upper limb; LLL: left lower limb; RLL: right lower limb; ATPO: anti-thyroperoxidase antibodies; ATG: anti-thyroglobulin antibodies; ANA: antinuclear antibodies; ANCA: anti-neutrophil cytoplasmic antibodies; AMA: anti-mitochondrial antibodies; ND: not done. | | | | | | | | | | | | | | | | | | | | |
